# Clinical characteristics and prognostic analysis of primary extranodal non-Hodgkin lymphoma of the head and neck

**DOI:** 10.18632/aging.205726

**Published:** 2024-04-05

**Authors:** Jiamu Lv, Yining Jiang, Tingting Yu, Shengrui Gao, Wanzhong Yin

**Affiliations:** 1 Department of Otolaryngology Head and Neck Surgery, First Hospital of Jilin University, Changchun, China; 2 Department of Neurosurgery, First Hospital of Jilin University, Changchun, China

**Keywords:** clinical characteristics, extranodal non-Hodgkin’s lymphoma, head and neck neoplasms, head and neck neoplasms, primary, prognostic factor

## Abstract

Objective: Primary extranodal non-Hodgkin’s lymphoma (PE-NHL) of the head and neck is the second common site of extranodal lymphoma, accounting for approximately one-third of all extranodal non-Hodgkin’s lymphoma (E-NHL). However, in recent years, large-scale PE-NHL case studies in China and worldwide are rare and not comprehensive enough. This work analyzed the clinical manifestations, pathological features, immunophenotypes and diagnosis of PE-NHL, as well as the factors affecting the treatment and prognosis.

Methods: A retrospective study was performed on 74 patients who were diagnosed with head and neck PE-NHL and treated for the first time. The clinical manifestations, pathological features, and immunophenotypes were summarized, and the factors related to the treatment and prognosis were analyzed.

Results: The most common site of this disease was the Waldeyer’s ring, followed by the nasal cavity. Diffuse large B-cell lymphoma was the most common type, followed by extranodal NK T-cell lymphoma nasal type. The 1-year, 2-year, and 5-year progression-free survival (PFS) rates were 76.4%, 67.9%, and 59.3%. The 1-year, 2-year, and 5-year overall survival (OS) rates were 89.4%, 85.6%, and 63.2%. ECOG score ≥ 2, Ann Arbor stage III or IV and IPI risk stratification identifying patients as the high-risk group were independent risk factors affecting the OS of patients with PE-NHL of the head and neck.

Conclusions: The most common site of PE-NHL in these Chinese patients was the Waldeyer’s ring, but the incidence in the nasal cavity was higher than that reported in Western countries. Radiotherapy combined with chemotherapy had better efficacy than chemotherapy alone, and the prognosis depended on the ECOG score and clinical stage. IPI had a better prognostic value in patients in the high-risk group of head and neck PE-NHL.

## INTRODUCTION

Primary extranodal non-Hodgkin’s lymphoma (PE-NHL) of the head and neck is a type of non-Hodgkin’s lymphoma (NHL) in which the tumor is mostly located in an extranodal site and is initially diagnosed in the head and neck, with or without the involvement of the contiguous lymph nodes at the time of diagnosis [1]. Studies have shown that the prevalence rate of PE-NHL in the head and neck region was 44.62%, the incidence of extranodal lymphoma accounted for 36.66% of all head and neck lymphomas [2]. PE-NHL of the head and neck is the second common site of extranodal lymphoma after the gastrointestinal tract, accounting for approximately one-third of all extranodal non-Hodgkin’s lymphoma (E-NHL) [3, 4].

The etiology and pathogenesis of PE-NHL of the head and neck are still unclear. Previous studies showed that the incidence is related to some factors, such as virus infection history, including Epstein-Barr virus, human herpesvirus-8, and human T lymphocytic leukemia virus type I [5, 6], history of immune-related diseases, such as rheumatoid arthritis, Sjogren’s syndrome and celiac disease, as well as history of radiation exposure, immunosuppression, congenital immunodeficiency, acquired immunodeficiency syndrome, and exposure to certain medications (including digoxin, phenytoin, and chemotherapeutic agents) [7, 8].

Foreign studies showed that the Waldeyer’s ring is the most commonly involved site, followed by the salivary glands and oral cavity [9–12]. However, in China, PE-NHL seems more common in the nasal cavity and sinus than in the oral cavity and salivary glands [13, 14]. So far, the most common NHL subtypes in developed countries are diffused large B-cell lymphoma (DLBCL) (approximately 30%) and follicular lymphoma (FL) (approximately 20%), while the incidence of other NHL subtypes is less than 10% [5]. Extranodal NK T-cell lymphoma nasal type (ENKTCL-NT) is the most common NK T-cell lymphoma in Asia and Latin America, accounting for 10% of NHL and 30% of extranodal lymphoma. ENKTCL-NT is also the most common histological type in China, compared to other lymphoma subtypes [15, 16]. Chemotherapy is the most common treatment strategy, followed by radiotherapy and immunotherapy [17]. PE-NHL of the head and neck is often manifested as swelling at the primary site accompanied by pain or neck mass formation, with no other specific clinical manifestations that may characterize the different histological types. Complicated pathological features make the early clinical diagnosis difficult, thus, the delayed diagnosis leads to a delayed treatment, consequently leading to the progression of the disease. Patients with advanced PE-NHL of the head and neck have a poor prognosis, which ultimately leads to a decline in overall survival (OS) and poor prognosis.

Therefore, this article summarized the clinical characteristics of patients with PE-NHL of head and neck treated in our hospital, with the aim of guiding the clinical work to a certain extent and reducing the failure in the diagnosis or misdiagnosis of this disease. Patients follow up was performed to determine the OS and PFS. The clinical manifestations, pathological features, immunophenotypes and diagnosis were summarized, and the factors related to the treatment and prognosis were analyzed.

## MATERIALS AND METHODS

The data of 74 patients with PE-NHL of the head and neck hospitalized at the First Hospital of Jilin University between February 2012 and February 2022 were collected and retrospectively analyzed. The deadline for follow-up was March 1, 2022 or when the patient died. Including gender, age, general state, clinical features, site of onset, endoscopic manifestation, laboratory examination, imaging examination, pathological and immunohistochemical results, treatment, as well as efficacy and survival status. The diagnosis was confirmed by pathological examination after endoscopic biopsy by electronic nasolaryngoscopy or surgical excision biopsy. The site of the primary lesion when multiple sites of the head and neck were involved was determined according to the location of the lesion center in imaging, the sequence of symptoms, the patient’s medical history. We collected the data of patients who received initial treatment in our hospital. According to the pathological and immunohistochemical results of these patients, the diagnosis of NHL was confirmed. Patients with incomplete medical records, other malignant tumors and serious life-threatening diseases were excluded.

According to the current PE-NHL standards in China, patients with an extensive involvement in and outside the lymph node are classified as intra-node lymphoma according to the different degree of involvement. The primary extranodal lymphoma was associated with more extranodal invasion (> 75%) and less intraductal involvement (< 25%). The primary lymphoma originated in the Waldeyer’s ring was associated with the extranodal lymphoma. All cases were classified according to the *2016 WHO Classification Guidelines for Lymphocytic neoplasms* [18] as follows: (1) Mature B-cell lymphoma: DLBCL, FL, mantle cell lymphoma (MCL), mucosa associated lymphoid tissue lymphoma (MALT) and Burkitt lymphoma (BL); (2) Mature NK T-cell lymphoma: ENKTCL-NT, peripheral T-cell lymphoma not otherwise specified (PTCL-NOS), angioimmunoblastic T-cell lymphoma (AITL) and subcutaneous panniculitis T-cell lymphoma (SPTCL). Appropriate antibodies were selected for immunohistochemical examination of paraffin sections by automatic immunohistochemical apparatus. The positive standard of bcl-2 was ≥70%. The positive standard of CD10, bcl-6 and MUM-1 was ≥30% [19]. According to Han’s classification, it can be divided into GCB type and non-GCB type [20]. The stage in each patient was assessed using the *Ann Arbor system* (developed in 1971 for Hodgkin’s lymphoma and later for non-Hodgkin’s lymphoma) [21]. Patients were divided into group A and B according to the presence or absence of B symptoms and systemic B symptoms, which include the presence of unexplained weight loss (10% in 6 months), and/or unexplained fever, and/or night sweat [22].

### Statistical analysis

Statistical analysis was performed using SPSS version 23.0 software program (SPSS Inc., Chicago, IL, USA). Chi-square test was used to compare the morphological differences among different pathological types. A value of p < 0.05 was considered statistically significant. The Kaplan-Meier survival analysis was used to perform the univariate survival analysis of the prognosis, the log-rank method was used to perform the significance test, and the Cox regression model was used to perform the multivariate analysis of the prognosis. A value of p < 0.05 was considered statistically significant.

## RESULTS

### Clinical and pathological characteristics

A total of 74 patients newly diagnosed with PE-NHL of the head and neck were involved in this study and were divided into 41 males and 33 females, with a male to female ratio of 1.24:1. The median age was 58.5 years with a range from 5 years to 87 years. Of the 74 patients, 4 cases (5.4%) were under 21 years old, 11 cases (14.9%) were between 21 and 40 years old, 29 cases (39.2%) were between 41 and 60 years old, and 30 cases (40.5%) were over 60 years old. A total of 66 cases (89.2%) had a ECOG score < 2 points, and 8 cases (10.8%) had a ECOG score ≥ 2 points. In addition, 49 cases (66.2%) were at stage I or II, and 25 cases (33.8%) were at stage III or IV according to the Ann Arbor stage. The clinical manifestations of PE-NHL of the head and neck were diverse, with different primary sites and pathological types, as well as varying symptom severity. Our statistical analysis revealed that 19 cases (25.7%) had an unexplained weight loss (10% in 6 months), and/or unexplained fever, and/or night sweat (B symptoms) manifested as weight loss and fever. Among the 74 patients, 33 cases (44.6%) had primary site swelling and pain, and 9 cases (12.2%) had neck mass. Pharyngeal pain accompanied by dysphagia and pharyngeal foreign body sensation were the most common clinical manifestations of NHL with the pharynx as the primary site, accounting for 18 cases (24.3%) and 14 cases (18.9%), followed by slurred speech (6 cases, 8.1%), dysphagia (5 cases, 6.8%), dyspnea (4 cases, 5.4%) and hoarseness (2 cases, 2.7%). When the nose and nasopharynx were the primary sites, the symptoms were nasal obstruction in 16 cases (21.6%), followed by runny nose (9 cases, 12.2%), nasal odor (4 cases, 5.4%), epistaxis (3 cases, 4.1%) and snot with blood (2 cases, 2.7%) (Table 1). The specific physical examination or endoscopy showed that the primary site swelling was accompanied or not accompanied by the formation of a mass. For example, PE-NHL of the tonsil was often presented as unilateral tonsil enlargement (16 cases, 21.6%), and only a few cases had bilateral tonsil enlargement (2 cases, 2.8%). In addition, the most common lesion morphology was a mass (53 cases, 71.6%), followed by local ulcer (11 cases, 14.9%), tumor formation with local ulcer (5 cases, 6.8%) and simple mucosal swelling (5 cases, 6.8%). The Chi-square test was used to compare the morphological differences of different pathological types, which revealed that the simple mucosal swelling was more common in mature NK T-cell lymphoma (*p* < 0.05), while there was no statistically significant difference in the morphology of other pathological types (Table 2).

**Table 1:** Clinical manifestations in PE-NHL of the head and neck.

**Clinical manifestations**	**Throat**	**Clinical manifestations**	**Nasal/nasopharyngeal**	**Clinical manifestations**	**Others**
	**N (%)**		**N (%)**		**N (%)**
Pain	18 (24.3%)	Nasal obstruction	16 (21.6%)	Symptom B	19 (25.7%)
Foreign body sensation	14 (18.9%)	Runny nose	9 (12.2%)	Neck mass	9 (12.2%)
slurred speech	6 (8.1%)	nasal odor	4 (5.4%)	Loose teeth	1 (1.4%)
Difficulty swallowing	5 (6.8%)	Nose bleeding	3 (4.1%)		
dyspnea	4 (5.4%)	snot with blood	2 (2.7%)		
Voice hoarse	2 (2.7%)				

**Table 2:** Pathological morphology of different pathologic types.

**Lesions form**	**Pathologic types**			**χ^2^**	***P***
	**Mature B-cell lymphoma**		**T and NK-cell lymphoma**		
Simple mucosal swelling	1 (1.92%)		4 (18.18%)	6.487	0.011^*^
Mass	39 (75.00%)		14 (63.64%)	0.093	0.761
Ulceration	8 (15.38%)		3 (13.64%)	0.037	0.847
Mass with ulceration	4 (7.69%)		1 (4.55%)	0.243	0.622

Among the 74 patients with PE-NHL of the head and neck, the pathological tissue was obtained by surgery or endoscopic biopsy (electronic nasolaryngoscopy). According to the WHO classification criteria proposed in 2016 [18], the 74 patients were pathologically classified into mature B-cell lymphoma (52 cases, 70.3%) and mature NK T-cell lymphoma (22 cases, 29.7%). The most common pathological type of PE-NHL of the head and neck was DLBCL, while the Waldeyer’s ring was the most common site, followed by ENKTCL-NT mainly occurring in the nasal cavity; other types were rare. The distribution of cases by histological type is shown in Table 3. Mature B-cell lymphoma included DLBCL (38 cases, 51.4%), FL (7 cases, 9.5%), MCL (4 cases, 5.4%), MAL T (2 cases, 2.7%), and BL (1 case, 1.4%). Mature NK T-cell lymphoma included ENKTCL-NT (16 cases, 21.6%), PTCL-NOS (4 cases, 5.4%), AITL (1 case, 1.4%), and SPTCL (1 case, 1.4%). The most common pathological type of PE-NHL of the head and neck was DLBCL. The specific relationship between pathological types and primary sites is shown in Table 3.

**Table 3:** Primary site and histopathological subtypes.

**Primary site**	**B-cell lymphoma**						**T and NK-cell lymphoma**				**Total**	**(%)**
	**DLBCL**	**FL**	**MCL**	**MALT**	**BL**		**ENKTCL-NT**	**PTCL-NOS**	**AITL**	**SPTCL**		
Waldeyer’s ring												
Tonsil	14	4	3					2			23	31.1
Base of tongue	6							1			7	9.5
Nasopharynx	1		1		1			1			4	5.4
Pharyngeal wall	2										2	2.7
Two sites or more	1						2				3	4.1
Nasal cavity	1			1			12			1	15	20.3
Paranasal sinus	3	1					2				6	8.1
Salivary gland												
parotid	2	1		1					1		5	6.8
submandibular	2	1									3	4.1
Oral cavity	2										2	2.7
Thyroid	2										2	2.7
Larynx	2										2	2.7
Total	38	7	4	2	1		16	4	1	1	74	100.0
(%)	51.4	9.5	5.4	2.7	1.4		21.6	5.4	1.4	1.4	100.0	

**Figure 1:**
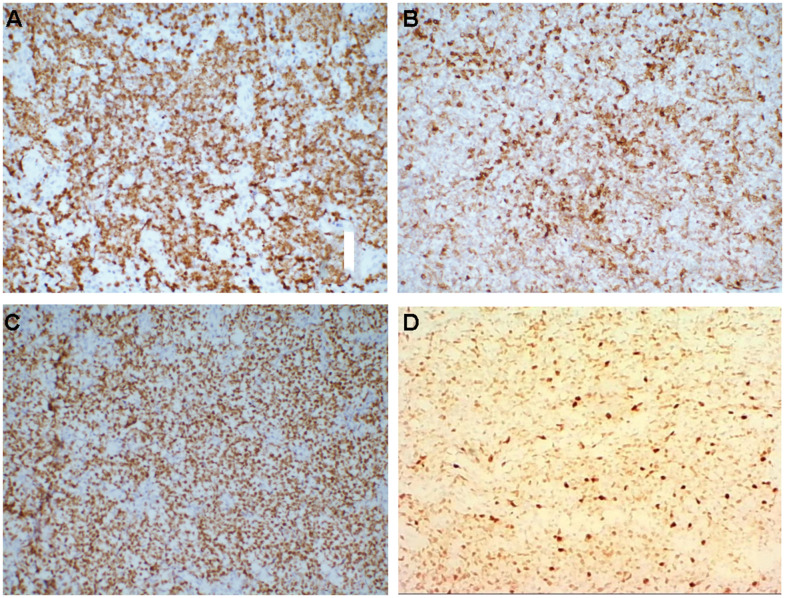
(**A**–**D**) Immunohistochemistry, medium magnification (×20), (**A**) Ki-67+, (**B**) Bcl-2+, (**C**) Bcl-6+, (**D**) MuM-1+.

According to the immunohistochemical results, the 74 cases were divided into 34 (45.9%) with Ki-67 < 60% and 40 (54.1%) with Ki-67 ≥ 60%. Bcl-2 positive cases were 49 (66.2%), Bcl-2 negative cases were 25 (33.8%). Among all the 74 patients, 38 had the pathological DLBCL type, which were divided into 16 (42.1%) with the GCB subtype and 22 (57.9%) with the non-GCB subtype according to Han’s classification (the GCB subtype include CD10+ or CD10 -, Bcl6+ and MUM1-; non-GCB subtype include CD10-, Bcl-6- or Bcl-6+, and MUM1+) (Figure 1). All laboratory and imaging examinations were completed and analyzed for all the 74 patients before treatment. The serum LDH level was normal in 52 cases (70.3%) and high in 22 cases (29.7%). β2 microglobulin was normal in 18 cases (24.3%) and was higher than normal in 56 cases (75.7%). All patients underwent whole-body PET/CT examination or MRI examination, including the 54 cases (73.0%) with lymph node involvement and the 20 cases (27.0%) without lymph node involvement.

In addition, the 74 patients were divided into low risk group (0~1 points) 35 cases (47.3%), low intermediate risk group (2 points) 19 cases (25.7%), high intermediate risk group (3 points) 15 cases (20.3%), and high risk group (4~5 points) 5 cases (6.8%) according to IPI [23].

### Treatment and efficacy evaluation

**Figure 2:**
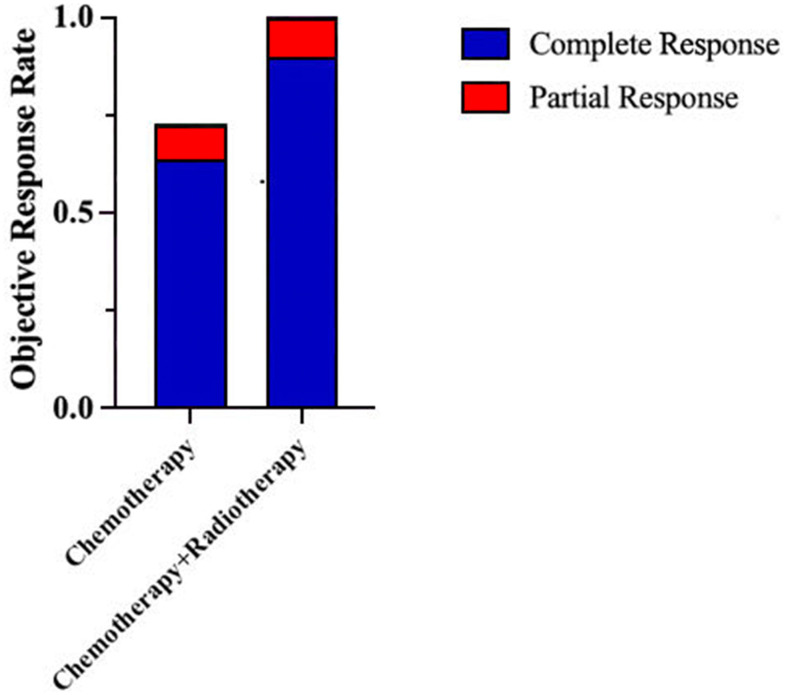
ORR of patients with PE-NHL of the head and neck under different treatment methods.

The treatment methods were divided into chemotherapy, chemotherapy combined with radiotherapy and surgery. Among them, 62 cases (83.8%) accepted chemotherapy alone, 11 cases (14.9%) accepted the combination of radiotherapy and chemotherapy and 1 case (1.4%) accepted the surgery. After treatment, 57 people were evaluated according to the efficacy; 39 cases (68.4%) achieved complete remission, 5 cases (8.8%) achieved partial remission, 7 cases (12.3%) had a stable disease, and 6 cases (10.5%) had a progressive disease. The objective response rate (ORR) was 77.2%. The ORR of the chemotherapy alone group was 72.3%, 30 cases (63.8%) achieved complete remission, 4 cases (8.5%) achieved partial remission. The ORR of the chemotherapy combined with radiotherapy group was 100.0%, 9 cases (90.0%) achieved complete remission, 1 case (10.0%) achieved partial remission (Figure 2).

### Survival and prognostic factors

**Figure 3:**
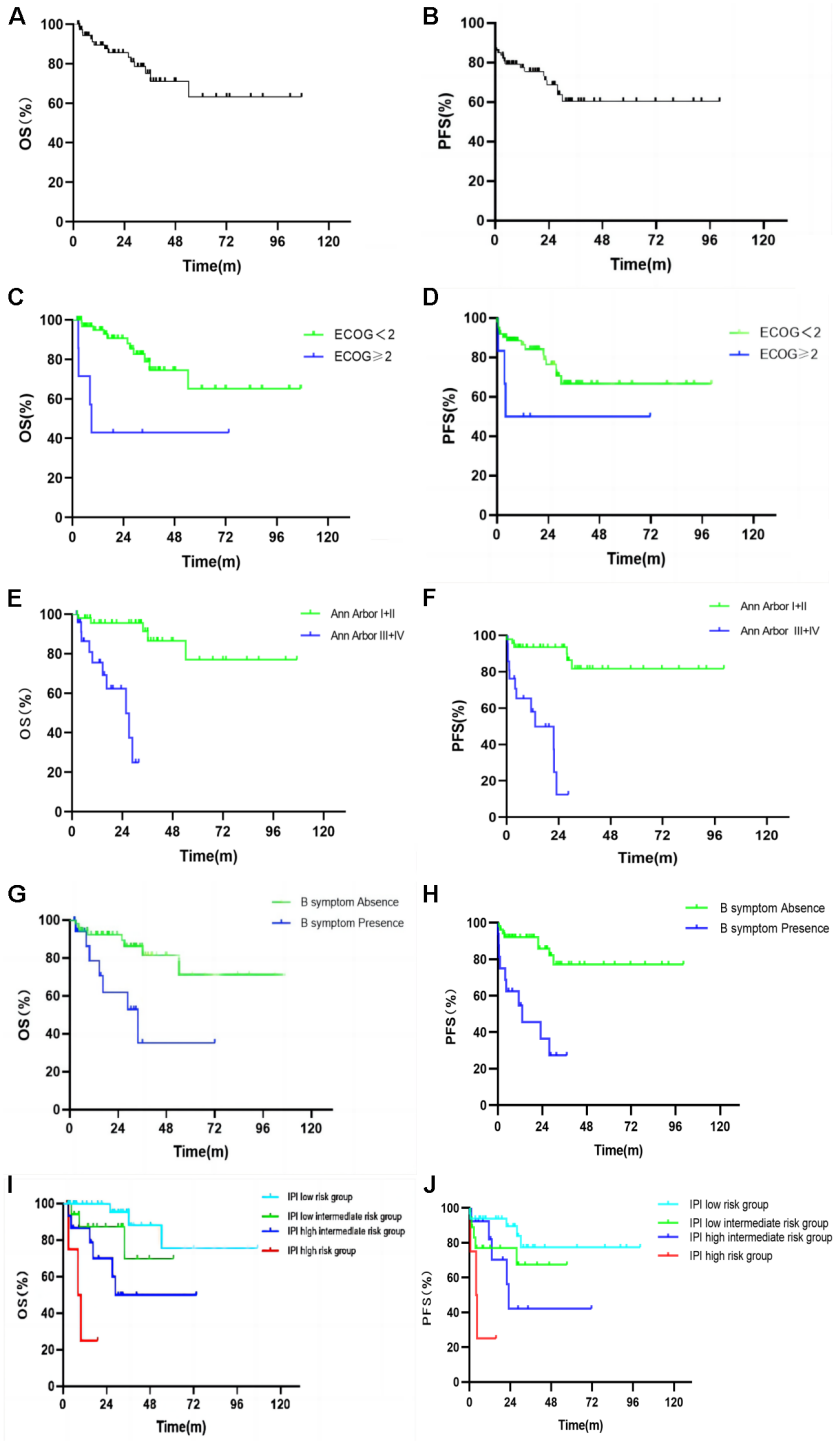
Kaplan Meier plots of the overall survival (**A**) and progression free survival (**B**) of all patients. Kaplan Meier plots of the overall survival and progression free survival by ECOG score (**C**, **D**), Ann Arbor stage (**E**, **F**), B symptoms (**G**, **H**), and IPI risk group stratification (**I**, **J**).

The follow up of the 74 patients was performed from February 2012 to March 2022, and 15 patients died, with a mortality rate of 20.3%. The median follow-up time was 24.4 months, median PFS was 17.5 months, and median OS was 24.6 months. The 1-year, 2-year, and 5-year PFS rate were 76.4%, 67.9%, and 59.3%, respectively (Figure 3A), while 1-year, 2-year and 5-year OS rates were 89.4%, 85.6%, and 63.2%, respectively (Figure 3B).

Kaplan-Meier survival analysis and Log-Rank test were used to analyze the factors that might influence the prognosis of PFS and OS in PE-NHL of the head and neck (Table 4). The median PFS of the group with ECOG score < 2 and that of the group with ECOG score ≥ 2 were 20.62 and 3.80 months (*p* = 0.01, Figure 3C), while the median OS was 27.17 and 8.63 months (*p* = 0.003, Figure 3D); the results were statistically significant (*p* <0.05). The median PFS of the Ann Arbor stage I or II group and III or IV group was 27.93 and 4.50 months (*p* <0.001, Figure 3E) and the median OS was 32.87 and 13.47 months (*p* <0.001, Figure 3F); the results were statistically significant (p <0.05). The median PFS of the normal LDH group and high LDH group was 19.25 and 9.67 months (*p* = 0.011). The median PFS between the group without B symptoms and the group with B symptoms was 21.93 and 5.80 months (*p* <0.001, Figure 3G). The median OS of the above groups was 27.17 and 14.67 months, respectively (*p* = 0.004, Figure 3H). The results were statistically significant (*p* <0.05). The median PFS of the IPI low-risk group, low intermediate risk group, high intermediate risk group and high-risk group was 26.27, 13.20, 12.13 and 4.50 months, respectively (*p* <0.001, Figure 3I) and the results were statistically significant (*p* <0.05). The median OS of the above groups was 31.80, 27.17, 18.73 and 9.77 months (*p* <0.001, Figure 3J), and the results were statistically significant (*p* <0.05).

**Table 4:** Univariate analysis for survival.

**Prognostic factors**		**N (%)**	**mPFS**	**P**	**mOS**	**P**
Age (y)	≤20	4 (5.4%)	55.05	0.156	62.63	0.085
	21-40	11 (14.9%)	7.90		13.03	
	41-60	29 (39.2%)	23.07		28.77	
	≥61	30 (40.5%)	7.95		15.02	
Gender	Male	41 (55.4%)	15.53	0.088	19.07	0.284
	Female	33 (44.6%)	19.03		28.30	
ECOG	<2	66 (89.2%)	20.62	0.010^*^	27.17	0.003^*^
	≥2	8 (10.8%)	3.80		8.63	
Pathological type	B-cell lymphoma	52 (70.3%)	19.25	0.547	26.50	0.236
	T/NK cell lymphoma	22 (29.7%)	12.80		17.42	
Ann Arbor stage	I+II (early stage)	49 (66.2%)	27.93	<0.001^*^	32.87	<0.001^*^
	III+IV (late stage)	25 (33.8%)	4.50		13.47	
LDH	Normal	52 (70.3%)	19.25	0.011^*^	26.50	0.192
	Elevated	22 (29.7%)	9.67		19.95	
β 2-MG	Normal	18 (24.3%)	14.42	0.653	23.52	0.995
	Elevated	56 (75.7%)	17.48		24.60	
Neck lymph node status	Absence	20 (27.0%)	15.56	0.115	23.12	0.109
	Presence	54 (73.0%)	18.75		24.60	
B symptom	Absence	55 (74.3%)	21.93	<0.001^*^	27.17	0.004^*^
	Presence	19 (25.7%)	5.80		14.67	
Ki-67	Ki-67<60%	34 (45.9%)	14.67	0.077	22.32	0.351
	Ki-67≥60%	40 (54.1%)	19.25		27.17	
Bcl-2	Absence	25 (33.8%)	25.63	0.265	32.77	0.388
	Presence	49 (66.2%)	15.57		19.47	
Treatment modality	Chemotherapy combined with radiotherapy	11 (14.9%)	15.57	0.127	28.30	0.371
	Others	63 (85.1%)	18.03		23.37	
IPI risk group stratification	low risk	35 (47.3%)	26.27	<0.001^*^	31.80	<0.001^*^
	low intermediate risk	19 (25.7%)	13.20		27.17	
	high intermediate risk	15 (20.3%)	12.13		18.73	
	high risk	5 (6.8%)	4.50		9.77	

According to the Han’s classification, patients with the pathologic type DLBCL were divided into the GCB subtype group and the non-GCB subtype group, and the PFS and OS in the two groups was analyzed. The median PFS of the GCB subtype group and non-GCB subtype group was 35.35 and 10.30 months, respectively (*p* = 0.011), the results were statistically significant (*p* <0.05), while the median OS was 41.00 and 16.55 months, respectively (*p* = 0.037). The results were statistically significant (*p*<0.05) (Table 5). The multivariate analysis indicated that the ECOG score ≥ 2 (*p* = 0.019), Ann Arbor stage III or IV (*p* = 0.008) and IPI high-risk group (*p* = 0.021), were correlated with the prognosis of head and neck PE-NHL (Table 6).

**Table 5:** Univariate analysis for survival of DLBCL.

**Han’s classification**	**N (%)**	**mPFS**	**P**	**mOS**	**P**
GCB	16 (42.1%)	35.35	0.011*	41.00	0.037*
non-GCB	22 (57.9%)	10.30		16.55	

**Table 6:** Multivariate analysis for survival.

**Prognostic factors**	**OS**	
	**HR (95% CI)**	***P***
Ann Arbor stage III+IV (late stage)	13.469 (1.962, 92.482)	0.008^*^
ECOG (≥2)	6.205 (1.352, 28.476)	0.019^*^
B symptom (presence)	1.186 (0.314, 4.482)	0.802
IPI (low risk)		0.020^*^
IPI (low intermediate risk)	2.448 (0.204, 29.427)	0.480
IPI (high intermediate risk)	5.729 (0.537, 61.094)	0.148
IPI (high risk)	18.234 (1.558, 213.451)	0.021^*^

## DISCUSSION

The clinical manifestations of head and neck PE-NHL lack specific characteristics, and its complex pathological features lead to difficulties in early clinical diagnosis, which has many adverse effects on the clinical diagnosis and treatment, resulting in disease progression. Patients with advanced PE-NHL of the head and neck have poor prognosis, ultimately leading to poor prognosis. Our study showed that the number of patients diagnosed with PE-NHL of the head and neck increased year by year in recent years and this article aimed to explore the clinical manifestations, pathological features, immunophenotypes and diagnosis, and analyze the factors related to the treatment and prognosis in Chinese patients.

Our results showed that PE-NHL of the head and neck was more common in middle-aged and elderly patients, with a median age of 58.5 years. The incidence rate in male patients was slightly higher than that in female patients, with a male to female ratio roughly similar to that in previous studies in China and Western countries [9, 11, 13, 14].

The Waldeyer’s ring is the most involved site in the PE-NHL of the head and neck, followed by the nasal cavity. However, the incidence of this disease in the nasal cavity in Chinese patients is significantly higher than that reported in western countries [9–12]. Further analysis found that the most common site of this tumor in the Waldeyer’s ring was the tonsil, which was similar to previous studies [13].

Many clinical works showed that the manifestations of the head and neck PE-NHL lack specificity and diversity. PE-NHL of the head and neck can present as a primary site swelling with pain and mass. Pharyngeal pain and pharyngeal foreign body sensation are the most common clinical manifestations of NHL whose primary site is the larynx. Patients with lesions located in the nose and nasopharynx are mainly showing symptoms such as a common cold or sinusitis with intermittent nasal congestion and fluid or blood in the prodromal stage. In these patients with nasal congestion, nasal mucosa swelling, purulent odor runny nose, granuloma, erosion, ulcer, necrosis, nasal septum or cleft palate perforation can also appear in the active period, although the general situation is not yet critical. However, the nasal mucosa, cartilage, midline bone and adjacent tissues are severely damaged at the end stage, leading to local deformation, cachexia, and systemic failure [24]. In our study, the lesion morphology of PE-NHL in the head and neck is mainly masses, followed by local ulcers, masses with local ulcers and mucosal swelling. Specialist physical examination or endoscopy showed that the primary site swelling was accompanied or not accompanied by the formation of a mass. PE-NHL in the tonsil was usually manifested as unilateral tonsil enlargement, and only a few bilateral tonsil enlargements were observed [25]. Clinical symptoms can also be represented by toothache and tooth loosening [14, 26]. However, pharyngitis, sinusitis, tonsillitis and other inflammatory diseases as well as other otorhinolaryngology head and neck malignancy and other otorhinolaryngology diseases can also show the above clinical manifestations. Approximately the 25.7% of patients in our study had B symptoms, which were manifested as weight loss and fever, and this percentage was higher than that previously reported [13, 27, 28]. Therefore, middle-aged and elderly male patients with unilateral tonsillar enlargement with or without tumor formation, repeated swelling at the onset site, with pain, fever and weight loss should be aware of the possibility of having PE-NHL of the head and neck. Imaging examination and biopsy should be further improved for pathological examination to avoid the failure in the diagnosis or misdiagnosis or delayed treatment.

The diagnosis of PE-NHL of the head and neck depends on pathological and immunohistochemical tests that determine the specific histological subtypes [29]. In this study, PE-NHL of the head and neck was divided into mature B cell origin and mature NK T-cell origin according to the origin of the lymphoma cells, and the former was more common. In our study, the most common pathological type was DLBCL, followed by ENKTCL-NT. This pattern is different from previous studies in Western countries. The proportion of ENKTCL-NT patients in China is higher than that of the same group of patients in Europe and the United States, which is roughly the same as previous studies in China [15, 16]. According to the classification method proposed by Hans et al. [20], DLBCL of the head and neck is divided into GCB type and non-GCB type. Previous articles suggest that the vast majority of DLBCL in PE-NHL of the head and neck belong to the non-GCB subtype, and GCB patients have a better prognosis than those with non-GCB [30]. Excessive superficial sampling under endoscopy may lead to inflammatory pathological results, thus affecting the clinical diagnosis. Therefore, in clinical practice, sampling from multiple sites and in-depth sampling during biopsy can improve the diagnostic accuracy of high-risk patients with highly suspected PE-NHL of the head and neck or endoscopic local ulcer formation, and multiple biopsies can be performed when the diagnosis is not clear. In terms of immunohistochemistry, previous studies showed that the expression of Ki-67 and Bcl-2 protein is correlated with NHL survival [31, 32]. However, in this study, the Ki-67 antigen expression positive cell index and Bcl-2 protein expression were not associated with the prognosis of patients with head and neck PE-NHL. In our study, univariate analysis suggested that the prognosis of GCB patients was better than those with non-GCB.

The main treatments for PE-NHL of the head and neck include surgery, radiotherapy, and comprehensive treatment. Comprehensive therapy includes chemotherapy, targeted therapy and immunotherapy. Rituximab, as an anti-CD20 (+) monoclonal antibody, selectively depletes CD20 (+) B cells but does not deplete CD20 (-) cells. Rituximab has a good safety profile when used in combination with CHOP regimens (cyclophosphamide + doxorubicin + vincristine + prednisone), and induces responses in more than 90% of patients with indolent or aggressive lymphoma, improving the outcomes in high-risk and low-risk patients, with a small additional toxicity [33–36]. In this study, almost all patients with CD20 (+) as demonstrated by immunohistochemistry were treated with rituximab; thus, the effect of chemotherapy alone and rituximab combined with chemotherapy on the prognosis was not compared. Although rituximab combined with chemotherapy has been well applied in clinical practice, still some advanced patients cannot be cured by R-CHOP combination therapy. Therefore, it is recommended that advanced patients participate in appropriate clinical trials, and those with relapsing or refractory diseases can be subjected to transplantation of hematopoietic stem cells if they meet the requirements [26]. Previous studies believed that radiotherapy is the main treatment for MALT lymphoma, FL, and other E-NHL, and can also be used as consolidation treatment, rescue treatment or palliative treatment after systemic treatment [37–39]. However, in our study, no statistical difference was observed in the impact of radiotherapy on prognosis, which may be related to the small sample size of patients subjected to radiotherapy. The prognostic analysis of our results showed that the biopsy and tumor resection were not statistically different in the influence on prognosis, but surgery combined with radiation and chemotherapy resulted in a better ORR compared with the pure radiation and chemotherapy. When the lesions of head and neck PE-NHL are confined to a single organ, the impact of surgical resection on prognosis still requires further research. In addition, the treatment of relapse-resistant patients and the value of applying new drugs on patients with PE-NHL of the head and neck in recent years still need to be further explored.

The prognosis of head and neck PE-NHL is not only related to the treatment method. Our study showed that patients with higher LDH than normal, B symptoms, ECOG score ≥ 2, Ann Arbor stage III or IV, and IPI score in the high-risk group had poor prognosis. The last three were independent risk factors affecting the prognosis of patients with head and neck PE-NHL, while IPI had a good prognostic value in these patients in the high-risk group. Patients with ECOG score ≥ 2 have a poor prognosis, suggesting that patients with poor physical condition during the disease had a shorter survival time. Therefore, the patient’s physical condition should be carefully assessed at the initial diagnosis to better evaluate the prognosis in clinical practice. Ann Arbor stage III or IV patients had a poor prognosis, suggesting that patients with advanced disease had a significantly shorter survival time. Therefore, early diagnosis and treatment are crucial in the prognosis of patients with head and neck PE-NHL. In addition, the univariate analysis of the prognosis of 74 patients with head and neck PE-NHL showed that age had no correlation with the prognosis of these patients, and this result was consistent with the results of relevant studies performed in China in recent years [13, 14]. The reasons for this result remain to be explored. In conclusion, typical changes are often not observed under endoscopy due to the diversity of the clinical manifestations of PE-NHL of the head and neck. Therefore, imaging examination, pathological examination and immunohistochemical examination should be further improved for suspicious cases. Among them, pathology and immunohistochemical examination are the main approaches in the diagnosis of PE-NHL of the head and neck. The efficacy and prognosis of this disease are affected by a variety of factors. Thus, it is necessary to distinguish head and neck PE-NHL from other malignant tumors in clinical practice to avoid delayed diagnosis and treatment. In general, the role of surgery is limited to open biopsy to determine the pathological and immunohistochemical typing. However, previous studies showed that a small number of patients can be treated with surgical resection or radiotherapy when some inactivated lymphomas are confined to a single organ [40]. In this study, only 1 case of indolent lymphoma confined to the submandibular gland was found, who received a simple surgical treatment and had a good prognosis. Postoperative review of PET-CT showed no residual lesions, which was consistent with previous studies. This study is a retrospective analysis from a single institution and single center, with a small sample size, incomplete data and incomplete database, resulting in limitations in the analysis of the results. The analysis of prognostic factors is not comprehensive enough, not including the patient’s socioeconomic status, and whether there are other malignant tumors or diseases that may affect survival. The above shortcomings will be improved in our future works.

The lack of specificity in clinical manifestations and complex pathological features of head and neck PE-NHL lead to certain difficulties in early clinical diagnosis, delayed treatment and affected patient prognosis. In the past decade, the prognosis of head and neck PE-NHL has been different with the deepening of doctors’ understanding of the disease, the continuous improvement of treatment methods, and the increasing patients’ health concern and treatment compliance. Currently, there is no unified prognostic evaluation system for PE-NHL of the head and neck in China and worldwide. Therefore, it is still necessary to conduct a large number of retrospective studies to clarify the prognostic factors of head and neck PE-NHL, so as to better guide clinical work. In addition, the prognosis of patients with advanced or refractory relapses is still poor, and its etiology, pathogenesis and prognostic factors need to be further studied. Multidisciplinary cooperation is needed to improve the diagnosis and treatment of head and neck PE-NHL, and individualized treatment plan should be developed according to the characteristics of each patient, so as to improve the diagnosis and treatment level and improve the prognosis of patients.

## CONCLUSIONS

The most common site of PE-NHL in these Chinese patients was the Waldeyer’s ring, but the incidence in the nasal cavity was higher than that reported in Western countries. The most common pathological type was diffuse large B-cell lymphoma (DLBCL). Non-germinal center B-cell like (GCB) type was more common and the prognosis was worse than that of GCB patients. Radiotherapy combined with chemotherapy had better efficacy than chemotherapy alone, and the prognosis depended on ECOG score and clinical stage. IPI had better prognostic value in patients in the high-risk group of head and neck PE-NHL.
